# RETRACTION: Delanzomib, a Novel Proteasome Inhibitor, Sensitizes Breast Cancer Cells to Doxorubicin‐Induced Apoptosis

**DOI:** 10.1111/1759-7714.70346

**Published:** 2026-07-10

## Abstract

**RETRACTION**: WangM. , LiangL. , LuJ. , YuY. , ZhaoY. , ShiZ. , LiH. , XuX. , YanY. , NiuY. , LiuZ. , ShenL. and ZhangH. , “Delanzomib, a Novel Proteasome Inhibitor, Sensitizes Breast Cancer Cells to Doxorubicin‐Induced Apoptosis,” Thoracic Cancer 10, no. 4 (2019): 918‐929, 10.1111/1759-7714.13030.30883017 PMC6449274

The above article, published online on 18 March 2019 in Wiley Online Library (wileyonlinelibrary.com), has been retracted by agreement between the authors; the journal Editor‐in‐Chief, Tateaki Naito; China Lung Oncology Group; and John Wiley & Sons Australia, Ltd. The retraction has been agreed upon following concerns raised by a third party. An investigation identified a duplication between the MDA‐MB‐231 α‐tubulin bands and the MDA‐MB‐361 bands in Figure 2a. An additional duplication was identified between the MCF7 p53 bands and the MDA‐MB‐361 PUMA bands in Figure 6. Further duplications were identified between some MCF7 JNK bands in Figure 6 and bands published earlier in another article. The MDA‐MB‐231 JNK bands were also previously published in that same article by the same author group. The authors acknowledged the duplications and explained that the errors occurred inadvertently during figure preparation; they also provided some supporting data. However, due to the extent and nature of the duplications, the editors have lost confidence in the results and conclusions presented in this article.

**RETRACTION**: M. Wang , L. Liang , J. Lu , Y. Yu , Y. Zhao , Z. Shi , H. Li , X. Xu , Y. Yan , Y. Niu , Z. Liu , L. Shen and H. Zhang , “Delanzomib, a Novel Proteasome Inhibitor, Sensitizes Breast Cancer Cells to Doxorubicin‐Induced Apoptosis,” Thoracic Cancer 10, no. 4 (2019): 918‐929, 10.1111/1759-7714.13030.30883017 PMC6449274

The above article, published online on 18 March 2019 in Wiley Online Library (wileyonlinelibrary.com), has been retracted by agreement between the authors; the journal Editor‐in‐Chief, Tateaki Naito; China Lung Oncology Group; and John Wiley & Sons Australia, Ltd. The retraction has been agreed upon following concerns raised by a third party. An investigation identified a duplication between the MDA‐MB‐231 α‐tubulin bands and the MDA‐MB‐361 bands in Figure 2a. An additional duplication was identified between the MCF7 p53 bands and the MDA‐MB‐361 PUMA bands in Figure 6. Further duplications were identified between some MCF7 JNK bands in Figure 6 and bands published earlier in another article. The MDA‐MB‐231 JNK bands were also previously published in that same article by the same author group. The authors acknowledged the duplications and explained that the errors occurred inadvertently during figure preparation; they also provided some supporting data. However, due to the extent and nature of the duplications, the editors have lost confidence in the results and conclusions presented in this article.

